# Microbiota of the indoor environment: a meta-analysis

**DOI:** 10.1186/s40168-015-0108-3

**Published:** 2015-10-13

**Authors:** Rachel I. Adams, Ashley C. Bateman, Holly M. Bik, James F. Meadow

**Affiliations:** Plant & Microbial Biology, University of California Berkeley, Berkeley, 94720 CA USA; Biology and the Built Environment Center, Institute of Ecology and Evolution, University of Oregon, Eugene, 97403 OR USA; UC Davis Genome Center, University of California, Davis, Davis, 95616 CA USA; School of Biosciences, University of Birmingham, Birmingham, B15 2TT UK

**Keywords:** Bacteria, Built environment, High-throughput sequencing, Microbiome, Negative controls, Source-sink dynamics

## Abstract

**Background:**

As modern humans, we spend the majority of our time in indoor environments. Consequently, environmental exposure to microorganisms has important implications for human health, and a better understanding of the ecological drivers and processes that impact indoor microbial assemblages will be key for expanding our knowledge of the built environment. In the present investigation, we combined recent studies examining the microbiota of the built environment in order to identify unifying community patterns and the relative importance of indoor environmental factors. Ultimately, the present meta-analysis focused on studies of bacteria and archaea due to the limited number of high-throughput fungal studies from the indoor environment. We combined 16S ribosomal RNA (rRNA) gene datasets from 16 surveys of indoor environments conducted worldwide, additionally including 7 other studies representing putative environmental sources of microbial taxa (outdoor air, soil, and the human body).

**Results:**

Combined analysis of subsets of studies that shared specific experimental protocols or indoor habitats revealed community patterns indicative of consistent source environments and environmental filtering. Additionally, we were able to identify several consistent sources for indoor microorganisms, particularly outdoor air and skin, mirroring what has been shown in individual studies. Technical variation across studies had a strong effect on comparisons of microbial community assemblages, with differences in experimental protocols limiting our ability to extensively explore the importance of, for example, sampling locality, building function and use, or environmental substrate in structuring indoor microbial communities.

**Conclusions:**

We present a snapshot of an important scientific field in its early stages, where studies have tended to focus on heavy sampling in a few geographic areas. From the practical perspective, this endeavor reinforces the importance of negative “kit” controls in microbiome studies. From the perspective of understanding mechanistic processes in the built environment, this meta-analysis confirms that broad factors, such as geography and building type, structure indoor microbes. However, this exercise suggests that individual studies with common sampling techniques may be more appropriate to explore the relative importance of subtle indoor environmental factors on the indoor microbiome.

**Electronic supplementary material:**

The online version of this article (doi:10.1186/s40168-015-0108-3) contains supplementary material, which is available to authorized users.

## Background

The microorganisms in, on, and around our bodies comprise a large portion of the biodiversity we encounter in our lives. Our microbial associates impact our health, both positively and negatively. Understanding the processes that structure indoor microbial communities is an important endeavor, since we spend the bulk of our time indoors and likely exchange many of our microbial passengers with various indoor habitats. In recent years, the scientific community has begun to recognize the importance of characterizing such human-associated habitats, with increasing numbers of studies seeking to determine the biodiversity, ecology, and public health implications of microbial assemblages present in the built environment. Investigations to date have included assessments of public restrooms [1, 2], hospitals [3–7], residences [8–18], university classrooms and office buildings [5, 19–22], artisan cheesemaking facilities [23], athletic facilities [24, 25], museums [5, 26], metropolitan subways [27–29], and even the evolutionary context of microbes indoors [30].

These individual studies typically target a specific location in order to elucidate the interrelationship between microbial communities and various environmental factors. Most recently, community assemblages have been described by targeting short fragments of ribosomal RNA (rRNA) genes and noncoding regions, such as the 16S rRNA gene in bacteria and archaea and the internal transcribed spacer (ITS) region in fungi. Gene fragments are amplified and sequenced from the pool of species present in environmental DNA samples, followed by data analyses that describe the community assemblage observed within a sample (*α*-diversity) as well as how much diversity is shared between different samples (*β*-diversity).

There is both promise and peril in combining separate studies into a meta-analysis. Individual efforts to characterize the microbes we encounter in buildings have shown that geography [10], building design and ventilation [31, 32], and occupant presence and activity [19, 20, 31, 32] can all contribute as drivers of indoor microbial communities. On the one hand, concurrent evaluation of these—and additional—studies has the potential to reveal large-scale biological trends and patterns in microbial community composition. On the other hand, there are several recognized limitations [33], with the dominant question being—can we detect true biological differences over the background of technical variation that exists across studies? In the case of microbial datasets, technical variation is defined as differences caused by experimental protocols, including but not limited to differences in the nature of the samples, DNA extraction methods, PCR amplification protocols, target genetic locus, sequencing primers, and sequencing platform. Differential experimental protocols can introduce significant bias and potentially obscure any meaningful biological patterns that may be observed across studies.

In this study, we conducted a meta-analysis of publicly available microbial datasets utilizing high-throughput sequencing (454, Illumina platforms) to investigate community patterns in the built environment. Additionally, supplementary datasets representing potential “source habitats” (human microbiome, outdoor environments) were analyzed alongside datasets from the indoor environment. Specifically, we aimed to address the following questions: (1) Are there consistent mechanisms evident across studies (biogeography, building operations, etc.) structuring microbial communities indoors? (2) Can we identify consistent source habitats for microbial communities in the built environment? (3) Are fungal and bacterial communities structured by the same processes across studies?

## Results and discussion

We compiled high-throughput 16S rRNA sequence data from 23 different studies, including 16 studies from built environments and seven from potential source environments, such as soil, the human microbiome, and outdoor air. We found that targeted biological comparisons were generally successful when using subsets of studies that shared a common experimental approach. Source tracking identified air and, to a lesser extent skin, as sources for indoor air, although for many samples, the sources were unidentified. In making comparisons across the entire set of studies, it was apparent that every dataset considered was unique in some aspect of habitat, sampling protocol, DNA extraction and amplification method, target 16S region, sequencing platform, or resulting sequencing depth and quality. Although our analysis approach mirrored that of other recent meta-analyses [33–35], unlike other studies, we found that technical variation across built environment studies overshadowed biological variation. This strong study-level effect was consistent across adjustments to the analysis pipeline. We conclude by discussing these findings in context of recommendations for future studies.

### Biological insights

#### Studies with shared habitats

We looked for subsets of studies that examined identical habitats across broad geographic areas. The common indoor habitats were limited to bathrooms and kitchens, so for this analysis, we included four studies: South Korea homes [11], Colorado restroom surfaces [1], Colorado kitchen surfaces [12], and North Carolina homes [9].

**Fig. 1:**
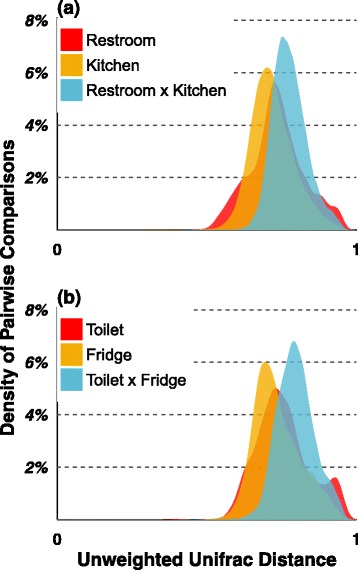
Bacterial community distance within and between indoor surfaces. A subset of studies from similar indoor environments was analyzed (Colorado kitchen surfaces, Colorado restroom surfaces, South Korea restroom and kitchen surfaces, and North Carolina kitchen and restroom surfaces), and figures show the density of unweighted UniFrac pairwise distances (**a**) within restrooms, within kitchens, and between restrooms and kitchen, as well as (**b**) within toilets, within fridges, and between toilets and fridges. Results indicate that the bacterial OTUs found on these surfaces tend to be more similar to each other than between surfaces

For the human microbiome, some evidence exists that the same body sites across individuals are generally more similar to one another than different skin sites within a single individual [36]. We hypothesized that, much like the human body, kitchens and restrooms were the surfaces that were likely to reveal consistent microbial community patterns, since both rooms are among the most likely to accumulate moisture on surfaces, and they receive similar periodic inoculation from microbe-rich sources (such as humans and food). As with skin, site-level similarity has been noted across residential surfaces for bacteria [1, 9, 12, 14]. For the subset of studies considered here, bacterial communities generally did reflect their respective surface (Fig. 1). For instance, regardless of the study, we found that communities from toilets were more similar to other toilets than to other surfaces in kitchens or restrooms. Each surface or room type was dominated by bacterial OTUs consistent with the most likely human-influenced source. For example, plant chloroplasts, presumably food-based, were the dominant type in kitchens, representing 17 % of sequences, while in the bathrooms, chloroplasts represented only 3.6 % of sequences. Conversely, skin-associated bacteria such as *Propionibacterium acnes*, *Corynebacterium*, and *Streptococcus* were dominant in the bathroom and less abundant than more environmental-associated bacteria *Acinetobacter* in kitchens, regardless of geographic location (South Korea, Colorado, and North Carolina).

#### Source tracking

**Fig. 2:**
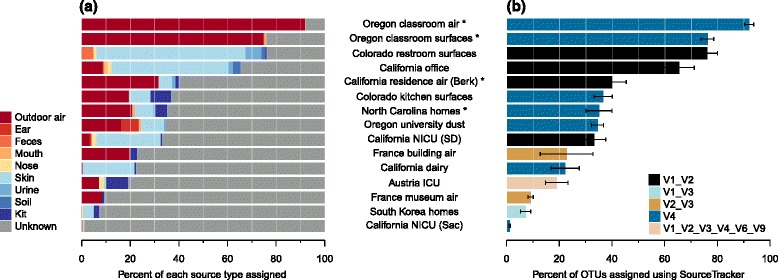
Sources tracking of indoor environments. A subset of samples from each of the studies (see Table 1) was analyzed using the SourceTracker algorithm to apportion microbial sources for different “sinks” of indoor settings. Prominent sources were outdoor air, skin, soil, and laboratory kits (**a**), although the likelihood of identifying sources varied strongly by study. **b** Those studies that were more likely to have sources identified were those that originally included source environment samples (using their own sampling and laboratory methods—denoted by *asterisk* in the figure), contained samples that were more strongly sourced from skin, or targeted the same variable region as those in the source samples

Source tracking is a Bayesian approach to estimate the proportion of a given “sink” community sample that is comprised of OTUs from a potential “source” sample [37]. For this study, sources were deemed to be outdoor air, soil, and human-associated samples (skin, feces, mouth, urine). Broadly, outdoor air and unidentified sources dominated the sources for indoor air environments (Fig. 2a); outdoor air averaged a mean proportion of 0.52 (range 0.003–0.98) while unknown averaged 0.43 (range 0.016–0.99). Skin was the next most identified source with a mean proportion of 0.03 (range 0–0.25). Indoor surface environments, compared to airborne assemblages, tended to be more strongly sourced from human-associated taxa, with an average proportion of skin of 0.17 (range 0–0.96), and outdoor air contributing a similar amount (0.14; range 0–0.95). In looking within indoor surface types, individual sources became more important. For example, urine and feces were observed to be a more dominant source in bathrooms compared to other areas (Fig. 2a). Thus, from the biological perspective, source tracking results largely support the intuitive understanding of environment representing the most common source populations for microbial taxa that get dispersed indoors. These results also largely mirror what has been shown in individual studies (e.g., [9, 14, 17, 19, 32]).

**Table 1:** Studies included in this meta-analysis

Study	Total no.	Target	PCR	Sequencing	DNA extraction	Avg. sequence	Avg % assignment	Sampling	Geolocation	Sampling	Sourcetracker	Citation	Data
name	of samples	region	primers	platform	method	length (bp)	of sequences	method		sites	assignment		source
	per study						per sample						
California	59	V1–V2	8f_357r	454 GS	Phenol:Chloroform	314	58.67 % (+/ − 13.79 %)	Air (settle	Albany, CA,	Rooms in apartment	Sink/source	[14]	Contributed
residence				FLX plus	MoBio PowerSoil			plate)	USA	building (living room,			by author
air (Berk) ^*a*^					DNA isolation kit					balcony, bedroom,			
										bathroom)			
California	86	V4	515f_806r	Illumina	ZR96 Fecal DNA	252	67.73 % (+/ − 14.76 %)	Surface	Davis, CA,	Rooms in industrial	Sink	[23]	Qiime
dairy ^*b*^				MiSeq	extraction kit			(cotton	USA	cheese processing			database
								swab)		building (wagon wheel			
										room, scooping room,			
										production room,			
										brine_main room,			
										bloomy_rind room,			
										drying room, aging			
										room, packaging room)			
California	147	V4	515f_806r	Illumina	ZR96 Fecal DNA	252	53.94 % (+/ − 10.22 %)	Surface	Sacramento,	Hospital (neonatal	Sink	[3]	Qiime
NICU (Sac) ^*b*^				MiSeq	extraction kit			(cotton	CA, USA	intensive care			database
								swab)		units) surfaces			
										and devices			
Colorado	285	V4	515f_806r	Illumina	MoBio PowerSoil	89	67.9 % (+/ − 27.1)	Surface	Boulder,	Kitchens in family	Sink	[12]	Qiime
kitchen				HiSeq2000	DNA isolation kit			(cotton	CO, USA	homes			database
surfaces ^*a*,*b*,*c*^								swab)					
Colorado	126	V1–V2	27f_338r	454 GS	MoBio PowerSoil	232	91.6 % (+/ − 7.46 %)	Surface	Boulder,	Public restrooms	Sink	[1]	Qiime
restroom				Junior	DNA isolation kit			(cotton	CO, USA				database
surfaces ^*a*,*b*,*c*^								swab)					
France	3	V2–V3	535f_789r	454 GS	QiaAmp	228	92.23 % (+/ − 1.86 %)	Air	Paris,	Hospital,	Sink	[5]	Contributed
building air				FLX				(filter)	France	museum,			by author
				Titanium						office building			
South Korea	28	V1–V3	9f_541r	454 GS	FastDNA SPIN	120	60.8 % (+/ − 17.68 %)	Surfaces	Seoul,	Rooms in	Sink	[11]	Qiime
homes ^*c*^				Junior	extraction kit			(Easy	Korea	family homes			database
								Swab Kit)		(kitchen,			
										bathroom)			
California	30	V1–V2	27f_338r	454 GS	DNeasy	221	94.22 % (+/– 4.67 %)	Surface	San Diego,	Hospital (neonatal	Sink	[7]	Qiime
NICU (SD)				FLX	Tissue Kit			(cotton	CA, USA	intensive care units)			database
								swabs)					
California	55	V1–V2	27f_338r	454 GS	DNeasy	227	93.95 % (+/ − 4.07 %)	Surface	San Francisco,	Office building	Sink	[22]	Qiime
office				FLX	Tissue Kit			(cotton	CA; New York,	objects			database
								swabs)	NY; Tucson, AZ				
Oregon	173	V4	515f_806r	Illumina	MO BIO	130	85.67 % (+/ − 7.83 %)	Dust	Eugene, OR,	University building	Sink	[47]	Contributed
university				MiSeq	PowerWater			(vacuum	USA	(office, storage,			by author
dust					DNA Isolation			filter)		classroom, food			
					Kit					service)			
North	384	V4	515f_806r	Illumina	directPCR	109	86.37 % (+/ − 15.91)	Surface (Rayon-	Raleigh-	Homes	Sink/source	[9]	Qiime
Carolina				HiSeq				tipped swab)	Durham Area,	(condominium,			database
homes ^*c*^				2000;					NC, USA	low-rise apartment,			
				Illumina						family homes)			
				MiSeq									
France	3	V2–V3	Custom	454 GS	Custom	263	87.42 % (+/ − 0.58 %)	Air	Paris, France	Art museum	Sink	[26]	Contributed
museum				FLX				(cyclone					by author
air				Titanium				sampler)					
Oregon	321	V4	515f_806r	Illumina	MO BIO	127	85.77 % (+/ − 5.54 %)	Air	Eugene, OR,	University	Sink/source	[19]	Contributed
classroom				MiSeq	PowerWater			(filter)	USA	classroom			by author
air ^*b*^					DNA Isolation								
					Kit								
Oregon	62	V4	515f_806r	Illumina	MO BIO	127	91.41 % (+/ − 4.00 %)	Surfaces	Eugene, OR, USA	University	Sink	[19]	Contributed
classroom				MiSeq	PowerWater					classroom			by author
surfaces ^*b*^					DNA Isolation								
					Kit								
Austria	24	V1–V2–	27f_1492r;	454 GS	Custom XS	236	94.66 % (+/ − 3.72 %)	Surfaces (BiSKits	Austria	Hopital	Sink	[6]	Contributed
ICU		V3–V4–	515f_927r	FLX	PC			and nylon-		(intensive			by author
		V6–V9; V4		plus				flocked swabs)		care unit)			
Connecticut	15	V3–V4	331f_797r	454 GS FLX	MoBio PowerMax	508	68.34 % (+/ − 3.52 %)	Vacuum (dust	New Haven,	University	N/A	[20]	Contributed
classroom air				Titanium	Soil DNA			and air), HVAC (air)	CT, USA	classroom			by author
					Extraction Kit								
Colorado	9	V1–V2	27f_338r	454 GS	MoBio UltraClean	225	83.38 % (+/ − 20.64 %)	Air (filter)	Steamboat	Aerial source	Source	[52]	Qiime
mountaintop air				FLX	PlantDNA				Springs, CO, USA	of biota			database
				Titanium	Isolation Kit					(atmosphere)			
Ireland	168	V4	520f_802r	454 GS FLX	QIAGEN kit	221	93.83 % (+/ − 3.16 %)	Surface	Cork, Ireland	Human gut (source)	Source	[53]	Qiime
elderly gut				Titanium				(feces sample)					database
Colorado	602	V1–V2	27f_338r	454 GS	MoBio PowerSoil	229	92.76 % (+/ − 4.56 %)	Surface	Boulder,	Human body	Source	[36]	Qiime
body sites				FLX	DNA isolation kit			(cotton	CO, USA	sites (source)			database
				Titanium				swab)					
Europe/Africa	29	V4–V5	784f_1061r	454 GS FLX	Custom	247	93.24 % (+/ − 3.17 %)	Surface	Boulpon, Burkina	Human gut	Source	[54]	Qiime
children gut				Titanium	proteinaseK			(feces Sample)	Faso; Florence, Italy	(source)			database
					phenolchloroform								
Colorado	182	V1–V2	27f_338r	454 GS FLX	MoBio UltraClean	228	94.78 % (+/ − 3.5 %)	Surface	Boulder, CO, USA	Human palms	Source	[43]	Qiime
undergraduate					PlantDNA Isolation Kit			(cotton swab)		(source)			database
palms ^a^													
N + S America	89	V1–V2	27f_338r	454 GS FLX	MoBio PowerSoil	231	47.4 % (+/ − 5.96 %)	Soil	USA, Puerto Rico,	Outdoor source	Source	[55]	Qiime
soils				Titanium	DNA isolation kit				Peru, Argentina,	of biota (soil)			database
									Canada				
Colorado	1071	V1–V2	27f_338r	Illumina GAIIx	MoBio PowerSoil	128	87.00 % (+/ − 15.42 %)	Surface	Boulder, CO, USA	Human and dog	Source	[56]	Qiime
family					DNA isolation kit			(cotton swab)		body sites (source)			database

We combined high-throughput sequence data from 23 published, publicly available studies characterizing the built environment (sink) or studies that characterize potential sources of microbial dispersal into the built environment habitat (source). The Sourcetracker method requires a priori assignment of the samples in each of the studies as either an ecological source or sink, as indicated in the Sourcetracker Assignment column; three studies included source samples in their study design

^a^Included kit controls

^b^Study also used in Open-reference OTU picking

^c^Shared habitats study

From the perspective of combining studies in meta-analysis, our results suggest that site-specific sources may be particularly important for air environments (Fig. 2b). Although limited in number, two studies of bacteria in indoor air also had outdoor air samples [15, 32], and one study of settled dust was also accompanied by localized outdoor source samples representing air [9]. For these studies, outdoor air accounted for a mean proportion of 0.59 compared to 0.14 for those studies without study-specific designed outdoor source samples. Another study conducted in the same building [19] as a previous study that did include specific outdoor air samples [32] also showed a high proportion of outdoor air as the source. Thus, generic outdoor air sources were less informative that site-specific ones, indicating that bacteria in outdoor air can be highly localized [15, 32]. Moreover, we also observed differences in the power of generic sources to identify sources depending on the target variable region (Fig. 2b). Overall, this exercise suggests that processing even a few comparable outdoor samples alongside built environment samples may be much more effective for accurately identifying sources of indoor microbes versus analyses relying on a more extensive set of outdoor samples from another study.

### Technical variation in indoor microbiome studies

**Fig. 3:**
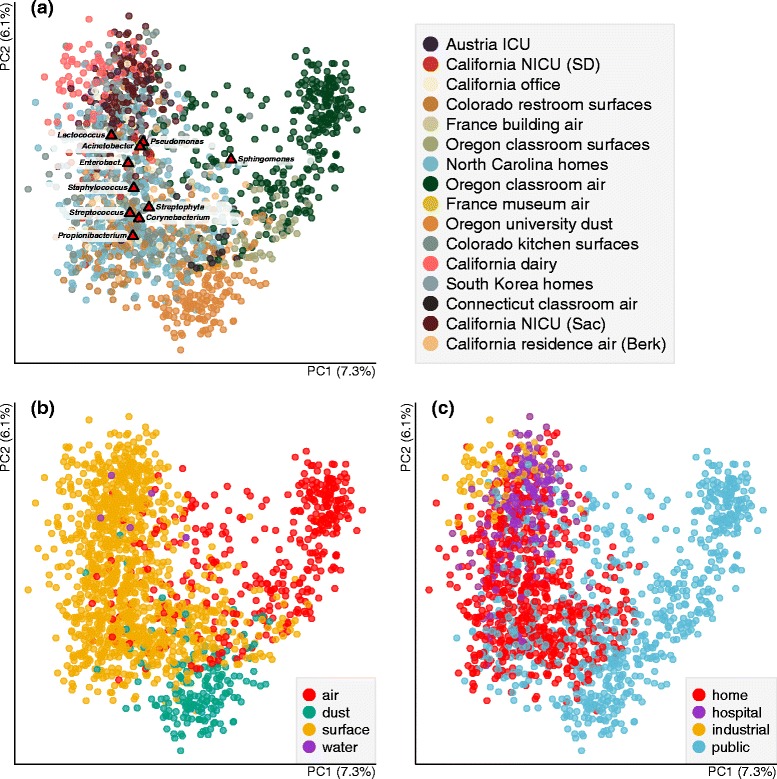
Principal coordinate analysis (PCoA) of bacteria in the 16 “sink” studies in this meta-analysis. Communities are compared using the unweighted UniFrac distance metric. **a** Studies cluster generally by study identity, and the top ten indicator taxa (*triangles*) are indicative of human-associated bacteria as well as outdoor-derived taxa. **b** Bacterial community composition also tend to group by the matrix type (the physical sample type) as well as the way the building is used (**c**)

When considering all studies together in principal coordinate analyses, a strong study effect is the most clearly discernible pattern, particularly when taxonomic-based metrics of ecological distance are used (Additional file 1: Figure S1) rather than a phylogenetically informed metric (Fig. 3a). While the results herein are presented based on UniFrac analysis, we discuss implications of this choice below. Clustering by study is perhaps unsurprising, since there are many opportunities for variations in sample collection in the built environment depending on the study question being addressed (Table 1). For example, across studies, samples were collected from such different building types as homes, classrooms, hospitals, and industrial settings. Collection material included swabs of surfaces, vacuumed floor dust, and air subjected to filtration. For studies using swab sampling, collections were from such varied surfaces as toilet seats, kitchen counters, and door trims, and the material of the swab itself also varied across studies. All measured experimental parameters had a significant effect on community composition. The factors with the largest explanatory power for bacterial communities were individual study (*R*^2^ = 0.4; Fig. 3a), geolocation, and specific sampling matrix (the physical sample type, e.g., dairy countertop versus toilet versus pillow, etc; *R*^2^ = 0.38), as well as general sampling matrix (Fig. 3b; source of the sequenced material differentiating air, surfaces, dust, and water; *R*^2^ = 0.18), and the use of the building (Fig. 3c; *R*^2^ = 0.17). Individual studies were generally carried out in a single location with a single type of sampling method, so these top most explanatory variables were correlated with each other. For example, individual study were linked to geolocation (*R*^2^ = 0.20) and building type (*R*^2^ = 0.49). Thus, while we were able to reveal biological variation within the built environment, the inter-study variation hindered our ability to identify consistent mechanisms (e.g., biogeography and specific building operations).

To explore the individual taxa driving patterns observed in the PCoA, we used bi-plots, which display the subset of bacterial taxa exerting the most influence over community clustering and separation patterns. The top ten taxa associatedm with indoor environments were recognizable as microbes associated with humans (e.g., *Corynebacterium, Streptococcus, Enterobacteriaceae, Staphylococcus, Propionibacterium, Lactococcus*) and outdoor habitats (e.g., *Streptophyta* [likely plant pollen], *Pseudomonas, Acinetobacter,* and *Sphingomonas*) (Fig. 3a).

### Implications of analysis workflow

Given the large differences in approaches to sampling the indoor environment that magnified technical variation across studies, we wanted to explore how choices in the analysis workflow might have influenced this outcome. Specifically, we discuss database representation, ecological distance metrics, and OTU picking strategy.

#### Database representation

**Fig. 4:**
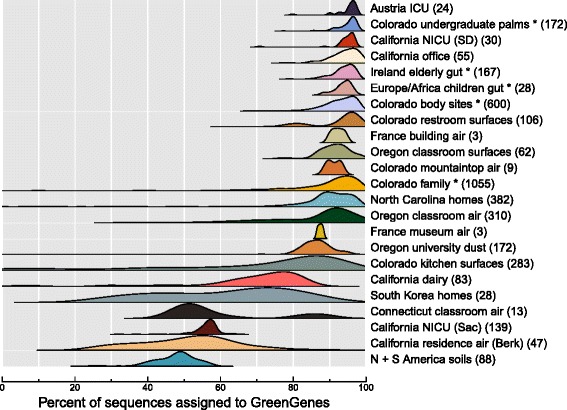
The proportion of sequences assigned to a reference database varies by study. Datasets from the human body (denoted by *asterisk* in the figure) and buildings with heavy dispersal from the human body tended to be better represented in the GreenGenes database, while soil and general outdoor sources were not as well represented. The numbers in parentheses show the number of samples in each study

We used closed-reference workflows to generate operational taxonomic units (OTUs) using the Greengenes database, since it is not currently possible to conduct *de novo* OTU picking on datasets that include separate 16S gene regions. However, this choice potentially introduces a consequential bias since studies varied in the percentage of sequences that matched the reference database (Fig. 4). For instance, human microbiome studies tended to be well represented (greater than 90 % assigned), while soils were very poorly represented (most samples were less than 50 %; see Table 1). This variation is not entirely surprising, since different environments are unequally represented in the GreenGenes database; a large scientific effort has focused on human-associated microbes in recent years, while in contrast, the genetic diversity present in most natural ecosystems remains largely uncharacterized. Most built environments in our meta-analysis ranged between these two extremes, which is to be expected since buildings include microbes sourced from both humans and outdoor environments to differing extents. While this study-level variation is interesting in itself, it may introduce a bias when using closed-reference OTU picking to compare across disparate studies and environments.

#### Choice of analysis metrics

Analysis of complex multivariate community data often necessitates the use of a distance or dissimilarity metric to compress many dimensions of variability into a single pairwise comparison. Four such metrics commonly used in sequence-based microbial community studies are Jaccard, Bray-Curtis, Canberra, and UniFrac (including both the weighted and unweighted variations) [38]. The first three are classical taxonomic metrics that assume equal relationships among organisms [39], whereas UniFrac incorporates and emphasizes the phylogenetic relationships among OTUs.

For many microbial community studies, taxonomic metrics can be desirable due to their ability to identify non-phylogenetic differences. For instance, the Jaccard metric can be evaluated as the simple proportion of taxa shared between two samples. The Bray-Curtis and Canberra metrics are both commonly used for their ability to prioritize shared distributions of the most abundant or rare taxa, respectively. The UniFrac metrics (weighted and unweighted), on the other hand, are heavily utilized in microbiome studies where broad-scale phylogenetic differences are important, such as when comparing the skin to gut microbiome where strong habitat differences favor entire functional groups or phylogenetic lineages. The trade-off is that a phylogenetic metric might miss out on subtle community differences when comparing very similar environments, while taxonomic metrics are inherently naive of functional community shifts.

**Fig. 5:**
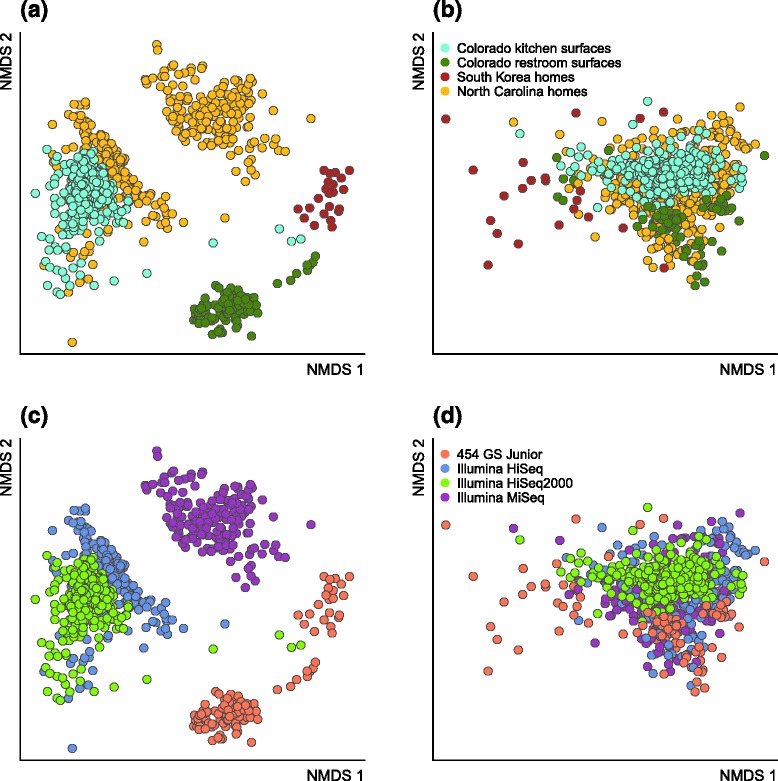
Difference between taxonomic and phylogenetic distance methods. Points from four studies of similar indoor environments (restroom and kitchen surfaces) are colored by study and analyzed by the Canberra community distance (**a**) and unweighted Unifrac (**b**). Similarly, points colored by sequencing protocols (including different primers and platforms) differ according to the Canberra distance (**c**) and the unweighted Unifrac (**d**)

Since the choice of distance/dissimilarity metric can be consequential, we compared the four metrics for their ability to overcome technical variation that results when disparate datasets are combined. Consistent with previous attempts [33–35], the unweighted UniFrac metric consistently succeeded in eliminating at least some of the technical variation in our dataset, while study-to-study variation was overwhelmingly evident when employing any of the taxonomic metrics. Although there is no clear method to determine how well a metric reduces study-to-study variation, we evaluated metrics based on two characteristics: (1) the extent to which individual studies overlapped with other studies from similar environments, such as when comparing surface communities on toilets (Fig. 5), and (2) the distribution of dissimilarity values (Additional file 2: Figure S2). This second criterion is crucial when comparing disparate studies with potentially very few OTUs in common, which results in many pairwise observations at or near 1, as opposed to the relatively normal distribution when using UniFrac. Given that the unweighted UniFrac clearly masked some important sources of technical variation (such as sequencing protocols in Fig. 5), it yielded a normal distribution of pairwise comparisons. Since our questions in the present study were constrained to broad phylogenetic patterns, we felt confident using the UniFrac metric for all analyses.

#### OTU picking strategy

**Fig. 6:**
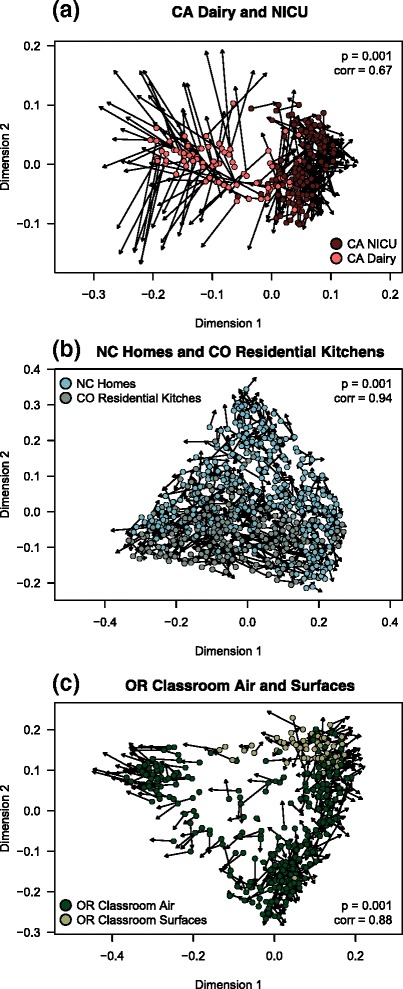
Closed and open-reference OTU picking yielded similar *β*-diversity results. Three pairs of studies were separately analyzed using the two OTU picking methods and then compared using Procrustes analysis. Each *point* is the result of open-reference OTU picking, and each *arrowhead* is the same sample from closed-reference OTU picking. A significant Procrustes statistic indicates that the results from *β*-diversity analysis are strongly correlated. The same sample across the two methods are linked with an *arrow*. **a** California dairy and neonatal intensive care unit, both near Davis, California; **b** North Carolina homes in and near Raleigh, North Carolina, and Boulder, Colorado residential kitchen surfaces; **c** Oregon classroom air and surface samples from Eugene. Although the California Dairy study appears to be different between the two methods (**a**), the Dairy site was statistically distinct from the paired NICU study regardless of OTU picking method

Qualitative conclusions based onto the two OTU-picking strategies were similar, in that patterns were generally consistent regardless of clustering method (Fig. 6). Moreover, the taxa indicative of each of the two environments overlapped between the two OTU-picking strategies. This exercise was useful for confirming that comparisons between studies appear to be robust to slight differences in analysis parameters.

By selecting pairs of studies to explore with open-reference OTU picking (see the “Methods” section), we were able ask whether certain taxa appeared to be associated with particular environments. The California dairy and California neonatal intensive care unit (NICU) did not show strong overlap in bacterial composition, and specific taxa seemed to be indicative of each dataset: *Lactococcus* and the salt-associated *Pseudoalteromonas* were two taxa linked to dairy samples, while the NICU was dominated more by human *Enterobacteriacea* and outdoor-associated bacteria *Acinetobacter*. Interestingly, an OTU of *Caulobacteraceae* was associated with the California NICU, and this bacterial family was also observed in a separate NICU study based in Pennsylvania [4]. Homes in North Carolina and kitchen surfaces in Colorado homes showed more overlap than the dairy and NICU. *Streptophyta*, *Enhydrobacter*, mitochondria, *Acinetobacter*, and *Pseudomonas* were more closely associated to kitchens while several human-associated groups including *Enterobacteriaceae*, *Streptococcus*, *Staphylococcus*, and *Corynebacterium* were found throughout homes. Like in homes, air and surfaces in an Oregon university classroom showed modest separation, with *Corynebacterium* and *Streptophyta* associated most strongly with surfaces while the air was more diverse, and linked to *Pedobacter, Microbacteriaceae, Sphingomonas, Oxalobacteraceae, Hymenobacter, Comamonadaceae,* and *Alicyclobacillus*.

### Kit controls

While previously utilized in some HTS studies from the built environment [1, 9, 12–16, 29], the use of kit controls to check for the potential introduction of contaminant taxa when carrying out standard molecular protocols is an increasingly recognized issue for environmental sequencing studies [40–42]. Recently, Salter et al. [41] showed that laboratory reagents and commercial DNA extraction kits can harbor their own distinct microbial communities, with the composition and diversity of the “kit microbiome” varying across manufacturer. Contaminant taxa can have a particularly large impact on low biomass samples (which are common in built environment studies), with kit-derived microbes effectively introducing a sampling artifact that drives the resulting *β*-diversity patterns observed across samples. By using technical controls during sample processing (concurrently sequencing blank DNA extractions alongside all samples), studies can identify and remove kit-associated taxa, thus reducing technical artifacts and helping to elucidate the true biological differences among samples [41]. For the studies included in this meta-analysis, we found that most did not include technical controls to profile kit-associated taxa, or at least did not make any such blank samples publicly available. Only four studies made use of negative controls [9, 12, 14, 43], out of the 23 data sets examined in total. All of these studies relied on the recovery of material from swabs. As an example, we explored the profile of the kit controls using these handful of studies that diligently provided such data, but we stress that the presence of contaminant taxa are not unique to these studies.

**Fig. 7:**
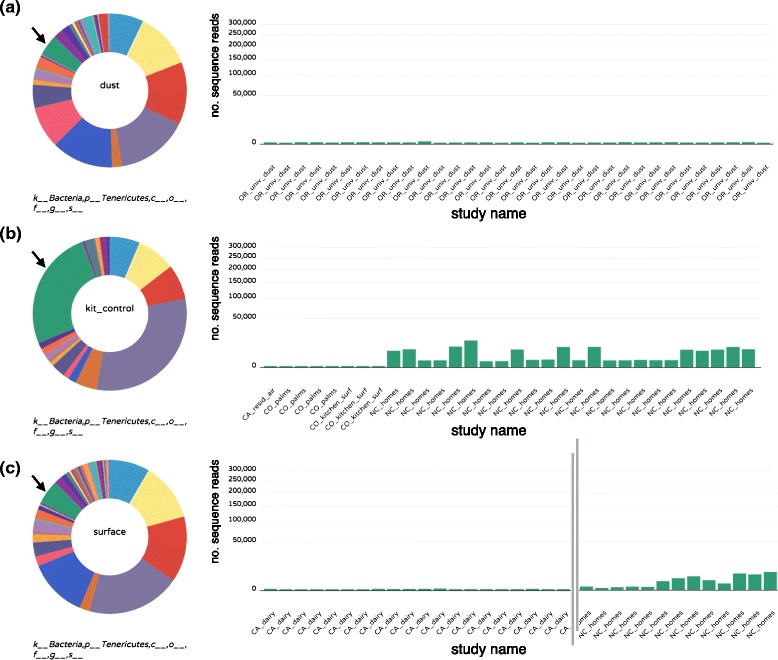
Example of taxonomic bias observed in technical control samples compared to environmental samples. The composition of each of the pooled **a** dust, **b** kit controls, and **c** surfaces samples is shown as a donut. The “kit microbiome” displayed higher abundances of the bacterial phylum *Tenericutes* (green slice in the donut chart indicated by arrows). Per-sample abundance of *Tenericutes* is represented by *green bars* displayed across all panels. Some samples in the North Carolina homes study showed similar levels of *Tenericutes* compared to the kit controls (**c**, *far right*); this implies some level of contamination in non-control environmental samples from this study, which the authors identified and removed in the original study [9]. *Donut* and *bar charts* were generated using the Phinch data visualization framework [63]

We observed that kit controls appeared to have a distinct microbial profile compared to other samples, with some taxa, such as the bacterial phylum *Tenericutes*, exhibiting significant enrichment in kit controls versus environmental samples (Fig. 7). Further inspection revealed that most OTUs assigned to the *Tenericutes* fell within the bacterial class Mollicutes or the genus *Mycoplasma*. Both of these groups contain well-characterized taxa representing ubiquitous, resilient laboratory contaminants of cell culture lines in particular [44, 45].

In contrast, the profile observed for other taxa such as Cyanobacteria (also potentially plant chloroplast sequences from pollen) showed an opposite trend, appearing abundant in dust samples while very few sequences from this taxon were found in the “kit microbiome” (Additional file 3: Figure S3). In the present meta-analysis, we used the results from technical controls to guide the downstream filtering of rRNA datasets and remove potential contaminating taxa, following the approach taken in [9]. While some entire taxonomic groups were easily removed due to their apparent contaminant status, such as *Tenericutes*, other taxonomic groups, such as *Corynebacterium* and *Staphylococcus*, were more difficult to remove since they are strongly human-associated (and could appear in kit controls due to “mistagging” of bar codes [46]), so removal could erroneously limit real biological insights. Notably, OTU picking strategy (open versus closed reference) generally did not increase the number of reads assigned to kit control samples (Additional file 4: Figure S4), suggesting contaminant taxa tend to be well represented in existing public databases and the kit microbiome can be sufficiently profiled even using closed reference workflows.

### Qualitative comparison of included studies

Given the limitations we encountered with compiling the raw data across studies, we explored whether general biological conclusions across studies were shared. This approach was qualitative, as quantitative metrics, such as individual bacterial diversity estimates, are incomparable when experimental protocols vary. Indoor surfaces are highly influenced by the nature of human contact [1, 9, 11, 19] and predictable human behavior; for example, bacteria on kitchen surfaces appear to be influenced by the introduction of food [12], while surfaces most often touched by hands contain high proportions of skin-associated bacteria [1, 6, 12].In non-residential settings, bacteria of outdoor origin may be a more influential source for surfaces, although human-associated microbes are still highly present [6, 19, 22]. Cleaning can reduce the human fingerprint [3, 9], and surfaces in industrial settings may show little influence of human contact [23]. The airborne bacteria found indoors also suggest a strong influence of human-associated bacteria as a source, in addition to outdoor-associated bacteria [14, 20, 26]. A longer sampling period of the air (on the order of weeks) demonstrates a shift towards human-associated bacteria in high-occupancy buildings [5, 20, 26], while shorter sampling periods (hours) with high outdoor ventilation suggest little effect of human occupancy on aerial bacterial composition indoors [32]. Features of building design, such as ventilation and connectedness of indoor spaces, can also cause predictable changes in the aerial bacterial communities found indoors [32, 47]. In the end, the broad results of our meta-analysis are generally consistent with this qualitative summary, but more resolved questions remain to be answered.

## Conclusions

High-throughput sequencing has vastly increased the quantity of data resulting from surveys of microbes across many different environments. The present study is a combined snapshot of studies in one such microbial habitat—our built environment—and reveals a scientific field that is still in its early stages. Geographic coverage of study locations tends to be focused around a few heavily sampled locations, with sparse representation elsewhere. Technological advances continue for high-throughput approaches (e.g., lengthening reads, increasing sequencing depth, new sequencing technologies), with methods and analysis protocols being continually updated even for a given sequencing platform (e.g., publication of new and updated bioinformatic workflows). Such rapid developments in high-throughput sequencing complicate the process of conducting meta-analyses, particularly for built environment studies. Buildings are rich in variation, differing by building materials, maintenance, use, and location. Moreover, sampling within the built environment relies on different collection matrices (surface, air, dust) and materials (swabs, filters, wipes, etc.), as the particular research questions dictate the most appropriate sampling techniques. For instance, the focus of a study could be on time-resolved samples of microbes indoors, in which case vacuum filtration onto filters would be more appropriate strategies than collection of settled dust. The varied nature of the building microbiome complicates efforts to standardize methods. As a consequence of this spectrum of variation, statistical power is effectively reduced, making it much harder to detect overarching biological differences. Taken together, these issues increase the difficulty of conducting meta-analyses for built environment studies, more so than other environments such as the human body and soils. Combining data sets is an alluring prospect, whereby the power of combined data may be greater than individual studies in terms of inferring biological patterns. Unfortunately, the present investigation demonstrates that meta-analysis of combined data sets is not as straightforward as we would like. Importantly, we emphasize that this exercise does not question the findings of any one particular study, as technical variation typically does not exist to the same extent within an individual study.

For future studies of the built environment, there are a number of ways to move forward. Firstly, the inclusion of appropriate negative (“kit”) controls is of paramount importance for quality control of sequence data, so that studies can filter and remove well-characterized contaminant taxa and prevent erroneous biological inferences based on artifacts. While some study-level variation that we observed is likely due to differences in experimental protocols details above, it is also likely that lab-specific contaminants also cause communities to diverge, and including kit controls offer a way to identify these spurious taxa. Secondly, there are many opportunities for individual indoor environment studies—with a common sampling strategy—to expand in scope: increasing the number of samples collected, including sampling of potential source habitats for indoor microbes, increasing the number and type of buildings surveyed, expanding the geographic focus if appropriate to address the study questions, and including experimental manipulation of the built environment in order to test specific hypotheses. Lastly, as this field matures, standardized data collection and description methods of operational building characteristics (e.g., [48, 49]) will allow for more meaningful comparisons across disparate studies.

As the health implications of the indoor microbiome are continually tackled by the research community (e.g., [50, 51]), understanding the basic factors that govern the potential pool of exposure is fundamental to modulating that environment. For example, it would be informative to be able to “rank” the mechanisms that structure microbial communities across different sampling types indoors. Specifically, how do biogeography, building function, ventilation type, and occupants and their activities interact to determine microbial composition, and how does that vary between airborne, dust, or surface microbes? Data presented here and elsewhere indicate that in the case of bathroom and kitchen surfaces, biogeography is less important than occupant activity, but source strengths are likely different for airborne microbes in those two residential locations. Moreover, it is unclear if the multitude of microbes identified in these different indoor environments are interacting (let alone alive), and how those interactions may affect its persistence in the built environment. Notable, fungi were excluded from full consideration in this meta-analysis due to limited study number, and it is unclear whether similar processes structure the two kinds of microbes. We are hopeful that as more built environment studies are conducted and made available, we will deepen our understanding of the global factors that structure and influence indoor microbial community assemblages.

## Methods

### Study inclusion criteria

We included studies if they met the following criteria: (1) published before May 15, 2014; (2) used high-throughput (HTS) amplicon sequencing to target 16S rRNA genes in bacteria/archaea or ITS rRNA in fungi; and (3) focused on built environments. We excluded several high-throughput studies due to low data quality compared to other studies (e.g., [13, 27, 31]), sequencing protocols that did not match the targeted single locus approach (e.g., [4]), and raw data that was not publicly deposited or otherwise obtainable (e.g., [8]). Table 1 describes some of the salient features of the 23 studies that met the above criteria and were thus included in the present meta-analysis [1, 3, 5–7, 9, 11, 12, 14, 19, 20, 22, 23, 26, 32, 36, 43, 47, 52–56].

Clone-based studies were not included in this study. In our search efforts for individual studies to include, we noted a large number of early built environment studies that relied on clone library construction followed by Sanger sequencing (e.g., [21, 57, 58]). While we acknowledge the potential contribution of such studies to a meta-analysis (providing an expanded set of geographical locations and a broadened survey of microbial diversity), preliminary exploration of these datasets indicated that clone libraries are unfeasible for inclusion with larger high-throughput datasets because of the orders-of-magnitude differences in sequence count and the fundamentally different laboratory protocols underlying such methods.

#### Preprocessing

Sequence datasets and sample metadata were either shared by the original authors, downloaded from public databases such as the NCBI Sequence Read Archive (SRA) or obtained from the QIIME online database, now superseded by the Qiita database (http://qiita.ucsd.edu).

Studies obtained from the QIIME database were downloaded as quality-filtered and demultiplexed datasets. For all other studies obtained from NCBI or the study authors, we implemented the same pre-processing quality filtering used for inclusion in the QIIME database, by setting parameters for the split_libraries.py (454 data) or split_libraries_fastq.py (Illumina data) scripts within QIIME version 1.8.0 [59]. Specifically, raw sequences from 454 Titatium and FLX platforms were excluded if not between 200 and 1000 nucleotides in length, had greater than six ambiguous bases, had homopolymer runs longer than 6 nucleotides, had mismatches in the primer, or could not be assigned to a sample using the barcode. For Illumina data, reads were truncated after more than three consecutive low-quality base calls and reads with <0.75 of the original read length remaining after truncation were subsequently discarded. Any reads containing ambiguous bases after quality trimming were also excluded. Because 454 Titanium chemistry yields longer read lengths, we trimmed all reads to the length achieved with standard FLX chemistry [33]. Pre-processed sequences from the QIIME database were then combined with sequences pre-processed in-house for downstream analysis.

#### Closed-reference OTU picking

Closed-reference operational taxonomic unit (OTU) picking followed the methods outlined in Lozupone et al. [33], using QIIME version 1.8. The closed-reference workflow is a database-dependent approach, using a pre-defined set of reference sequences with known taxonomy (the manually curated Greengenes database [60]) to cluster sequences into OTUs and assign taxonomy to environmental sequences. This approach is advantageous for comparing studies that target different 16S gene regions (e.g., V4 versus V9), since the underlying database is comprised of full-length gene sequences (≈1500 bp). However, in closed-reference OTU picking, taxonomic assignments are inherently constrained by the coverage of species and groups present in the reference database, and thus, results are limited to this subset of known microbial diversity. Closed-reference OTU picking was carried out using the pick_closed_reference_otus.py script with default parameters and the –enable_rev_strand_match flag. Identical sequences were first grouped using the prefix-suffix method implemented in QIIME, followed by clustering of representative sequences using UCLUST at 97 % sequence similarity against the reference sequences in the Greengenes database (May 2013 release). Sequences that did not have at least 97 % identity to any reference sequences were discarded. The average percent of sequences assigned per study is described in Table 1. Finally, taxonomy was assigned to each representative OTU sequence using the corresponding Greengenes hit, generating an OTU table containing the sample taxonomy, sequence counts per sample, and metadata from the 23 individual studies. Downstream data exploration and *β*-diversity analyses were carried out using QIIME and R.

Few of the 23 studies that we evaluated included laboratory control samples, such as sequencing blanks from DNA extraction kits and reagents. This is increasingly recognized as a necessary component for low-biomass, high-throughput sequencing studies [41]. Putative contaminant taxa were removed from the OTU table when an abundant taxonomic group was identified as originating from laboratory contamination (that is, the taxa appeared only in studies originating from a single laboratory), and thus, their presence added to laboratory-centric technical variation. Specifically, control samples from two studies that provided control samples [1, 9] informed that OTUs in the phylum *Tenericutes* and orders Oceanospirillales, Alteromonadales, EW055, and Tremblayales were likely contaminants. Overall, our determination of contaminant taxa were constrained by the lack of laboratory control samples from the majority of studies. More robust analysis would likely result in the removal of hundreds of OTUs from this analysis and thus improve study-to-study comparison. Data exploration revealed that overall conclusions do not change without removal of these putative contaminant taxa.

To account for substantial variation in sequencing depth among studies, the OTU table was rarefied to 1000 sequences per sample, which is often sufficient to draw *β*-diversity conclusions in a variety of environments [61, 62]. For studies where metadata permitted, we removed samples that were clearly source environments (e.g., outdoor air), and used these in a SourceTracker analysis [37] to identify sources of indoor microbes. In addition to QIIME [59], the Phinch data visualization framework [63] was used to explore processed and rarefied OTU tables in order to explore biological patterns and compare microbial communities observed across studies.

#### Open-reference OTU picking

Since closed-reference OTU picking inherently constrains the number of individual OTUs recovered from environmental studies, and thus potentially limits *β*-diversity resolution, we ran additional analyses on a subset of studies in order to identify potential biases introduced through OTU picking. A subset of six studies representing the V4 region of the 16S rRNA gene (the most common region analyzed across studies in this meta-analysis) were selected. We subdivided these studies into three pairs of datasets that utilized the same primer sequences and position on the 16S rRNA gene. The start and end positions of sequence reads within each dataset were confirmed by manually inspecting sequence alignments against the Greengenes 16S reference set. The final dataset pairs were (1) neonatal intensive care unit and dairy facility, both in California [3, 23], (2) two studies focused on the dust and air from an Oregon university classroom [19, 32], and (3) two studies of residential properties, one focused on North Carolina homes [9] and another focused on kitchens in Colorado [12]. For all dataset pairs, open-reference OTU picking was carried out using the pick_open_reference_otus.py script in QIIME, at 97 % sequence identity and 10 % subsampling of sequence reads not matching the Greengenes reference database. OTU picking was carried out using the –enable_rev_strand_match flag and default settings for all other parameters. Procrustes analysis was used to assess the influence of OTU-picking strategy on *β*-diversity inference.

#### Fungi

Fungal samples that relied on high-throughput sequencing in the built environment, at the time of analysis, were numbered at 469 across six studies (compared to nearly 4,000 samples across 23 studies for bacteria). Due to the limited number of samples and the limited overlap in community composition that was observed, fungi were not explored further (see Additional file 5: Text S1).

## Availability of supporting data

Full documentation of all scripts, commands, and parameters used for data analysis in this study are available on GitHub (https://github.com/jfmeadow/BEMAFinalAnalysis). Final taxa tables (in.biom format) and mapping files are also available from the GitHub site.

## Additional files

Additional file 1
**Figure S1.** Bray-Curtis ordination. Principal coordinate analysis (PCoA) of bacteria in the 16 “sink” studies using the Bray-Curtis taxonomic metric. Compare this to Fig. 3 a in the main text. The top ten indicator taxa are shown as triangles. (PDF 105 kb)

Additional file 2
**Figure S2.** Pairwise distance observations. Comparison of the distributions of ecological distances among taxonomic and phylogenetic metrics. *Y*-axes on the histograms (along the diagonal) indicate distribution density, and all other axes are unitless and are bound by 0 and 1. The distribution of UniFrac distances (bottom panel) is nearly normal compared to the taxonomic distributions that are skewed toward 1. This suggests that this choice of unweighted UniFrac is best suited for ordination and *β*-diversity analysis. The scatter plots (off the diagonal) show the correlation of values between the pairs of metrics, indicating that the different metrics are highly correlated with one another, and generally yield similar information. (PDF 3174 kb)

Additional file 3
**Figure S3.** *Cyanobacteria*. Example of taxonomic bias observed in technical control samples compared to environmental samples for taxa likely to be present in environmental samples. The composition of each of the pooled (a) dust and (b) kit controls is shown as a donut. The dust composition displayed higher abundances of *Cyanobacteria* (blue slice in donut chart indicated by arrows.) Per-sample abundance of *Cyanobacteria* is represented by blue bars displayed across all panels. Donut and bar charts were generated using the Phinch data visualization framework [63]. (PDF 146 kb)

Additional file 4
**Figure S4.** OTU picking strategy and kit controls. Comparison of OTU picking strategy on the sequences from the kit controls. Two studies were compared for the total number of sequence reads assigned in the kit controls, in closed-reference (a) versus open-reference (b) OTU picking workflows. One study, the North Carolina homes (24 control samples [9]), was remarkably similar in sequence reads assigned regardless of OTU picking method, while the other, Colorado kitchens (3 control samples [12]), was different. The relationship between the total number of assigned reads is shown in (c). (d and e) The taxonomic make-up of the samples is consistent at the phylum level between the two OTU picking strategies for one study but is different for another. (PDF 11 kb)

Additional file 5
**Text S1.** Fungal analysis. Description of the analysis steps for the fungal studies and basic findings, detailing why fungal studies were excluded from further analysis. (PDF 84 kb)
