# Supplementary material for: Microbiota of the indoor environment: a meta-analysis
Source: Microbiome. 2015 Oct 13;3:49. doi: 10.1186/s40168-015-0108-3 (PMC4604073; doi:10.1186/s40168-015-0108-3)
Supplement: Additional file 1 — Figure S1. Bray-Curtis ordination. Principal coordinate analysis (PCoA) of bacteria in the 16 “sink” studies using the Bray-Curtis taxonomic metric. Compare this to Fig. 3 a in the main text. The top ten indicator taxa are shown as triangles. (PDF 105 kb) [file 40168_2015_108_MOESM1_ESM.pdf]

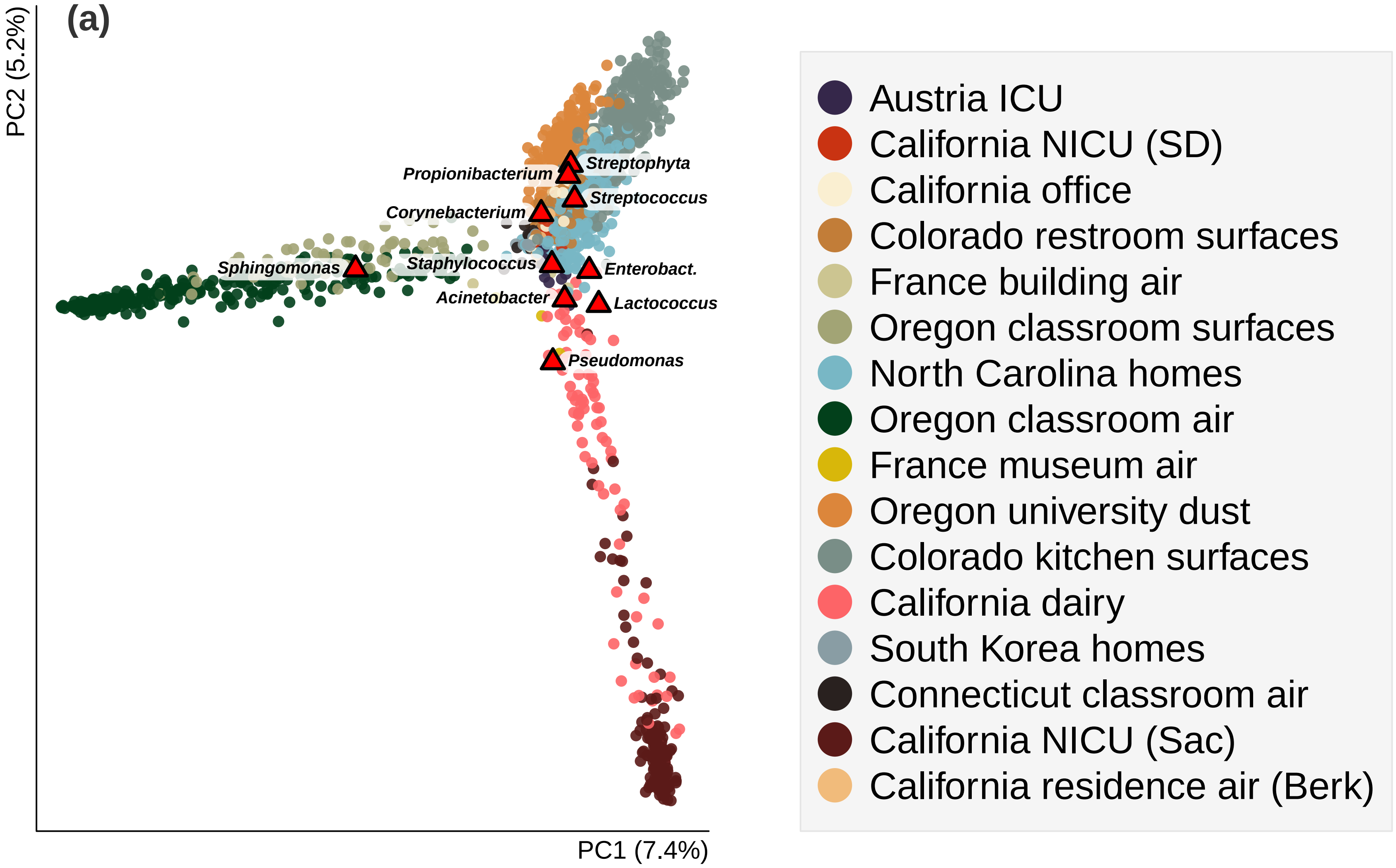

(a)
PC2 (5.2%)
PC1 (7.4%)
Streptophyta
Propionibacterium
Streptococcus
Corynebacterium
Sphingomonas
Staphylococcus
Enterobact.
Acinetobacter
Lactococcus
Pseudomonas
Austria ICU
California NICU (SD)
California office
Colorado restroom surfaces
France building air
Oregon classroom surfaces
North Carolina homes
Oregon classroom air
France museum air
Oregon university dust
Colorado kitchen surfaces
California dairy
South Korea homes
Connecticut classroom air
California NICU (Sac)
California residence air (Berk)
