# Supplementary figures and images for: Microbiota of the indoor environment: a meta-analysis
Source: Microbiome. 2015 Oct 13;3:49. doi: 10.1186/s40168-015-0108-3 (PMC4604073; doi:10.1186/s40168-015-0108-3)

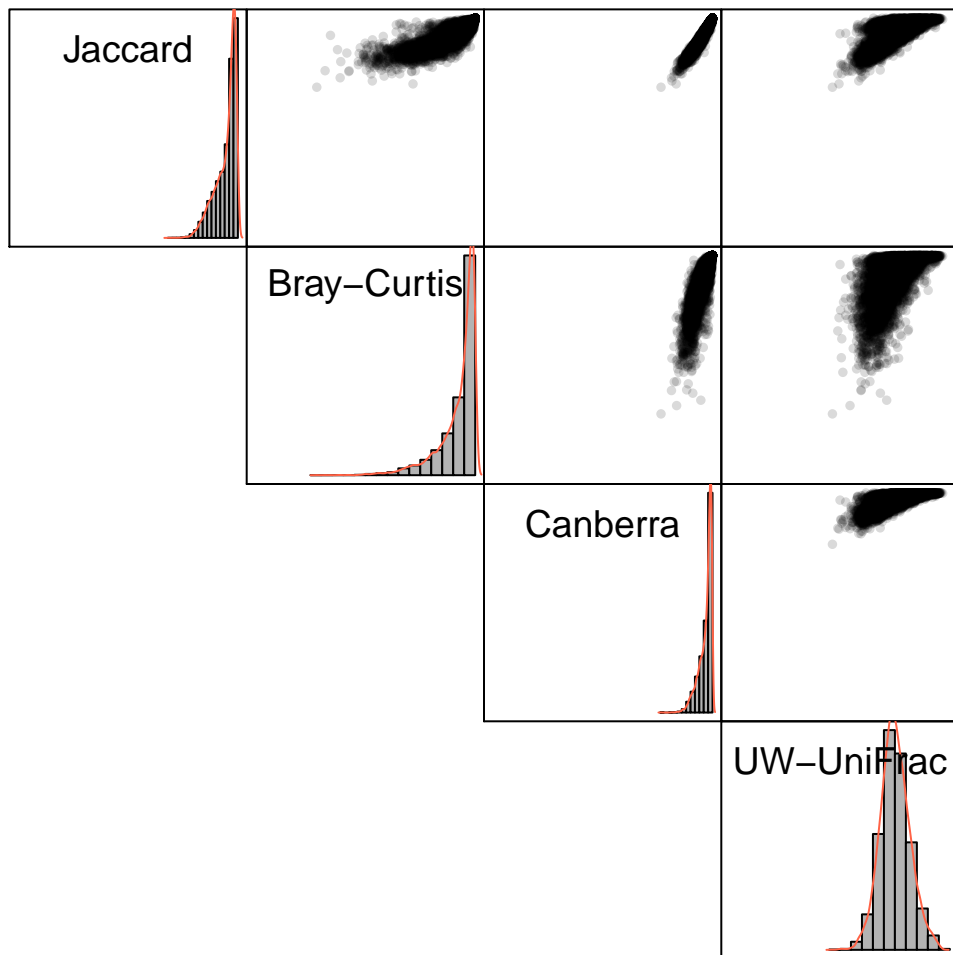

Supplement: Additional file 2 — Figure S2. Pairwise distance observations. Comparison of the distributions of ecological distances among taxonomic and phylogenetic metrics. Y-axes on the histograms (along the diagonal) indicate distribution density, and all other axes are unitless and are bound by 0 and 1. The distribution of UniFrac distances (bottom panel) is nearly normal compared to the taxonomic distributions that are skewed toward 1. This suggests that this choice of unweighted UniFrac is best suited for ordination and β-diversity analysis. The scatter plots (off the diagonal) show the correlation of values between the pairs of metrics, indicating that the different metrics are highly correlated with one another, and generally yield similar information. (PDF 3174 kb) [file 40168_2015_108_MOESM2_ESM.pdf]

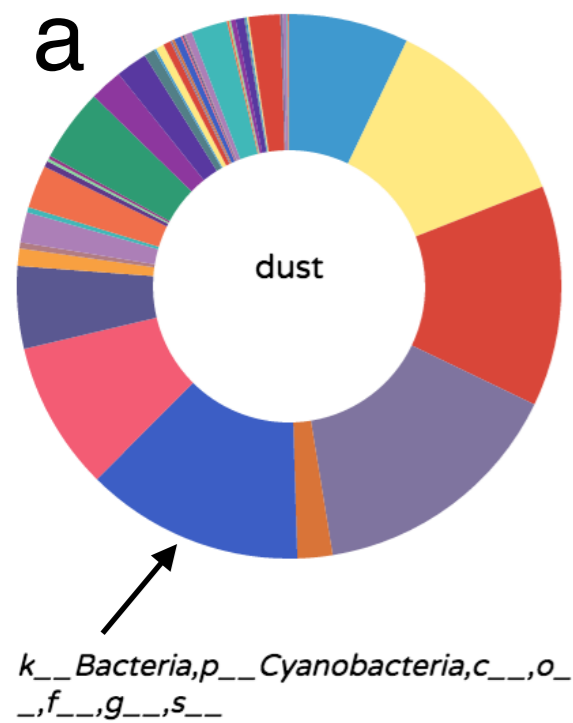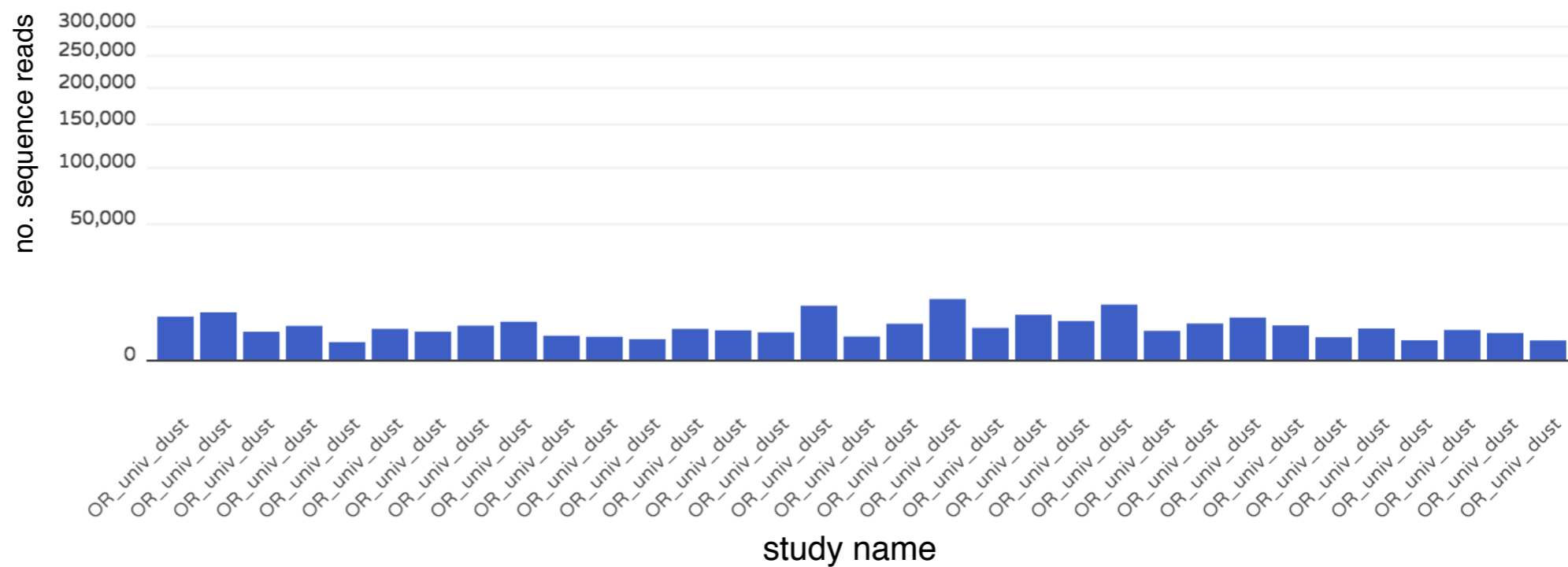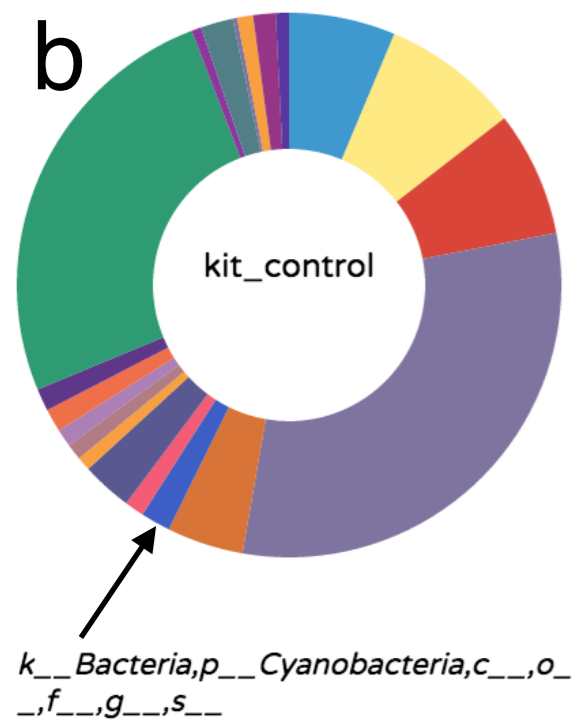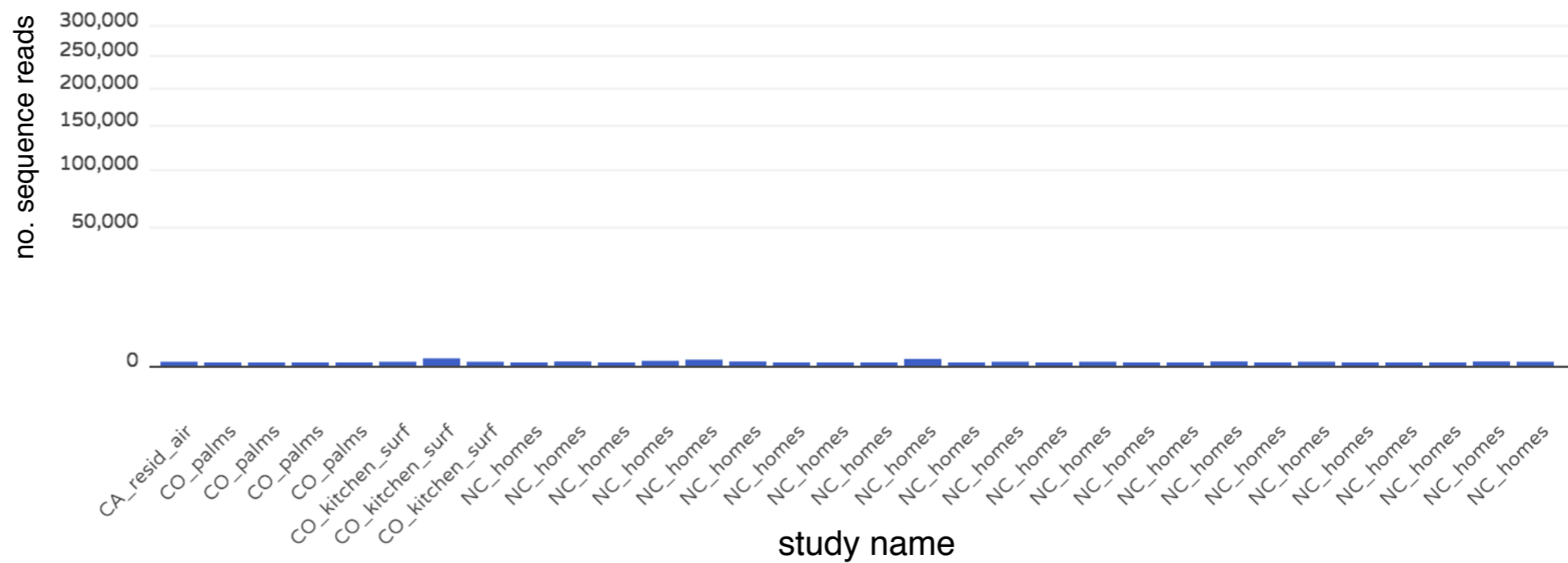

Supplement: Additional file 3 — Figure S3. Cyanobacteria. Example of taxonomic bias observed in technical control samples compared to environmental samples for taxa likely to be present in environmental samples. The composition of each of the pooled (a) dust and (b) kit controls is shown as a donut. The dust composition displayed higher abundances of Cyanobacteria (blue slice in donut chart indicated by arrows.) Per-sample abundance of Cyanobacteria is represented by blue bars displayed across all panels. Donut and bar charts were generated using the Phinch data visualization framework [63]. (PDF 146 kb) [file 40168_2015_108_MOESM3_ESM.pdf]

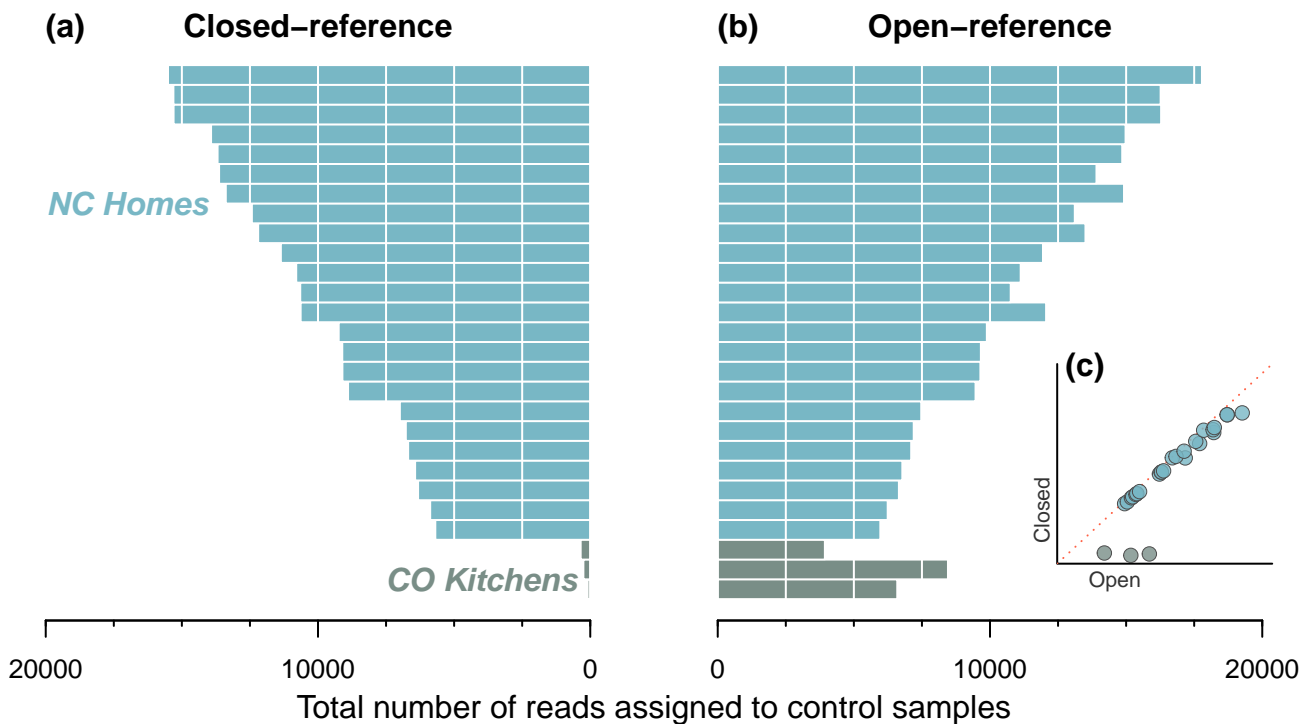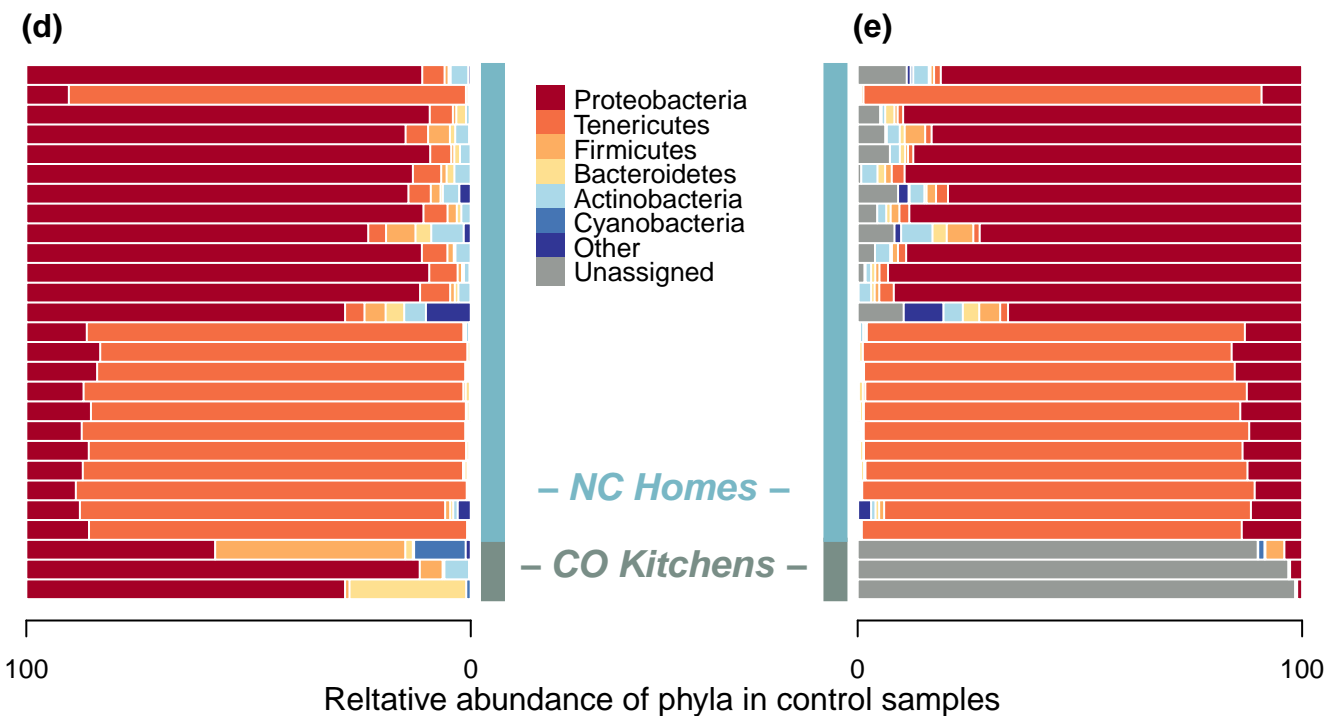

Supplement: Additional file 4 — Figure S4. OTU picking strategy and kit controls. Comparison of OTU picking strategy on the sequences from the kit controls. Two studies were compared for the total number of sequence reads assigned in the kit controls, in closed-reference (a) versus open-reference (b) OTU picking workflows. One study, the North Carolina homes (24 control samples [9]), was remarkably similar in sequence reads assigned regardless of OTU picking method, while the other, Colorado kitchens (3 control samples [12]), was different. The relationship between the total number of assigned reads is shown in (c). (d and e) The taxonomic make-up of the samples is consistent at the phylum level between the two OTU picking strategies for one study but is different for another. (PDF 11 kb) [file 40168_2015_108_MOESM4_ESM.pdf]
