# Supplementary material for: Microbiota of the indoor environment: a meta-analysis
Source: Microbiome. 2015 Oct 13;3:49. doi: 10.1186/s40168-015-0108-3 (PMC4604073; doi:10.1186/s40168-015-0108-3)
Supplement: Additional file 5 — Text S1. Fungal analysis. Description of the analysis steps for the fungal studies and basic findings, detailing why fungal studies were excluded from further analysis. (PDF 84 kb) [file 40168_2015_108_MOESM5_ESM.pdf]

Additional file 1: Text S1

Fungal analysis

There were six fungal studies that met our criteria: 1) sequence data available as of August 22, 2014; 2) used high-throughput (HTS) amplicon sequencing to target ITS rRNA in fungi; and 3) focused in built environments (Amend et al., 2010; Adams et al., 2013a; Adams et al., 2013b; Adams et al., 2013c; Dannemiller et al., 2014; Hoisington et al, unpublished). All studies relied on 454 pyrosequencing. Titanium reads were converted FLX length during processing of sff files ("-t" flag for process_sff.py), and reads had to have a minimum length of 150bps ("-l 150" flag for split_libraries.py). All reads across studies were combined for OTUs classification using the open reference OTU picking process within QIIME (pick_open_reference_otus.py). The reference database was a filtered and taxonomy annotated UNITE (Abarenkov et al., 2010; Bokulich and Mills, 2013). Prefiltering against the database was disabled as was the sequence alignment and tree-building steps ("-prefilter_percent_id 0.0 --suppress_align_and_tree"). The minimum OTU occurrence was two sequences ("--min_otu_size 2"), and all sequences that did not match with the reference database (i.e. subsampling at 100%, "-s 1" flag) were clustered *de novo* using the default uclust algorithm (Edgar 2010). For visualization of the relations between samples, the samples were rarefied to 100 sequences per sample, and community distance relied on the binary Jaccard index.

Two studies (Amend et al., 2010; Adams et al., 2013b) showed little overlap in community composition with other studies (see Supplementary Figure). We hypothesize that this has to do with low read quality compared to other studies (Adams et al., 2013b) and a different target region (the ITS2 locus) relative to all other studies (ITS1 locus) (Amend et al., 2010). While the remaining four studies showed overlap, this small number of studies also varied in sample processing such as sampling matrix, method, and location. While we set out to study whether the same environmental and ecological processes act to structure both bacterial and fungal communities in buildings, the small number of fungal studies available ultimately shifted our focus towards the more comprehensive datasets for bacteria/archaea.

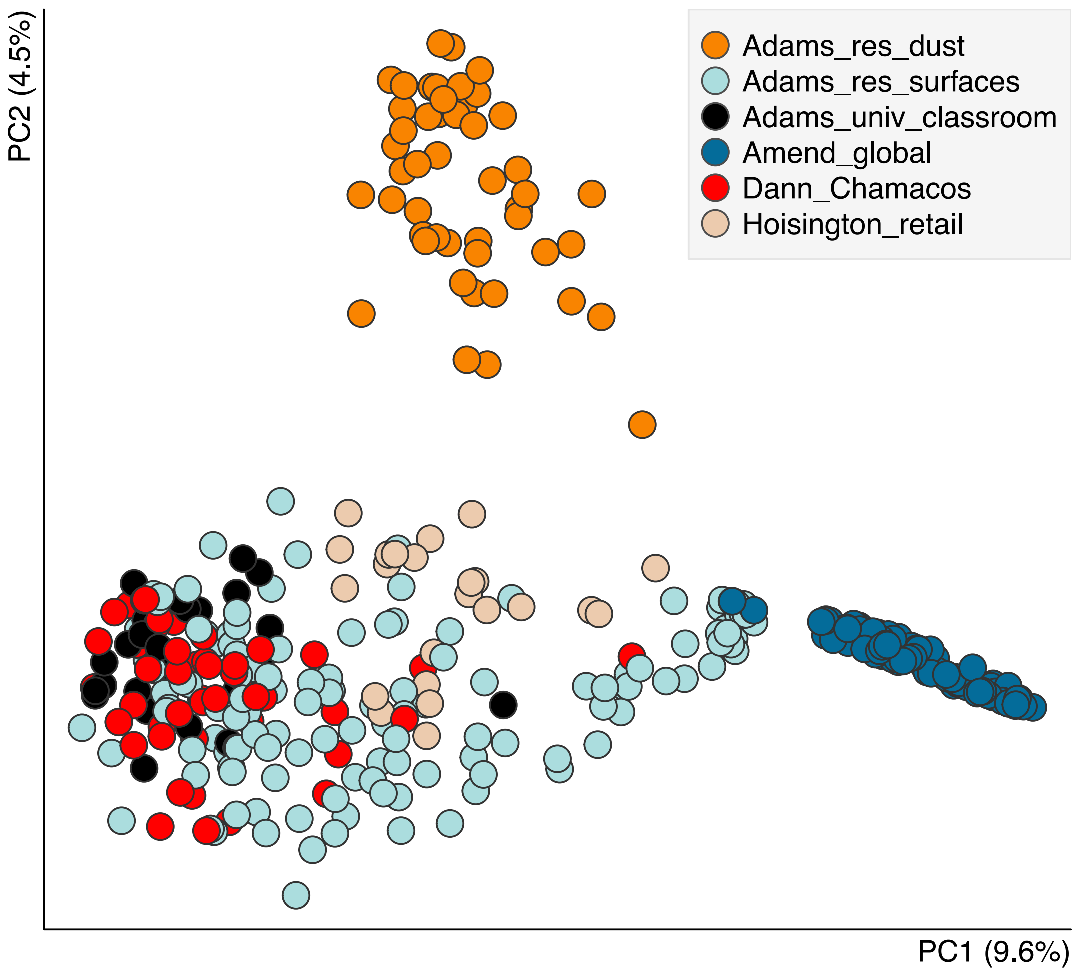

Abarenkov, K., Nilsson, R.H., Larsson, K.H., Alexander, I.J., Eberhardt, U., Erland, S., Hoiland, K., Kjoller, R., *et al.* (2010) The UNITE database for molecular identification of fungi — recent updates and future perspectives, *New Phytol.*, 186, 281-285.

Adams, R.I., Amend, A., Taylor, J.W. and Bruns, T.D. (2013a) A unique signal distorts the perception of species richness and composition in high-throughput sequencing surveys of microbial communities: a case study of fungi in indoor dust, *Microb Ecol*, 66, 735-741.

Adams, R.I., Miletto, M., Taylor, J.W. and Bruns, T.D. (2013b) Dispersal in microbes: Fungi in indoor air are dominated by outdoor air and show dispersal limitation at short distances, *ISME J.*, 7, 1262-1273.

Adams, R.I., Miletto, M., Taylor, J.W. and Bruns, T.D. (2013c) The diversity and distribution of fungi on residential surfaces, *PLoS ONE*, 8, e78866.

Amend, A.S., Seifert, K.A., Samson, R. and Bruns, T.D. (2010) Indoor fungal composition is geographically patterned and more diverse in temperate zones than in the tropics, *Proc. Natl. Acad. Sci. USA*, 107, 13748-13753.

Bokulich, N.A. and Mills, D.A. (2013) Improved selection of internal transcribed spacer-specific primers enables quantitative, ultra-high-throughput profiling of fungal communities, *Appl Environ Microbiol*, 79, 2519-2526.

Dannemiller, K.C., Mendell, M.J., Macher, J.M., Kumagai, K., Bradman, A., Holland, N., Harley, K., Eskenazi, B., *et al.* (2014) Next-generation DNA sequencing reveals that low fungal diversity in house dust is associated with childhood asthma development, *Indoor Air*, 24, 236-247.
